# Association of *Helicobacter pylori* infection with the correa cascade: a single-center, retrospective cohort study

**DOI:** 10.3389/fcimb.2026.1838434

**Published:** 2026-07-08

**Authors:** Zhangsen Huang, Liang Gu, Yuntao Shi, Songyao Chen, Songcheng Yin, Yilin Yang, Zhaowen Shi, Yujiao Zhong, Zhengli Wang, Xin Chen, Hai Li, Changhua Zhang, Yulong He

**Affiliations:** 1 Digestive Medicine Center, The Seventh Affiliated Hospital of Sun Yat-sen University, Shenzhen, China; 2 Guangdong Provincial Key Laboratory of Digestive Cancer Research, The Seventh Affiliated Hospital of Sun Yat-Sen University, Shenzhen, Guangdong, China; 3 Digestive Medicine Center, Fengqing people’s Hospital, Lincang, China; 4 Department of Gastroenterology, Fengqing County Hospital of traditional Chinese Medicine, Lincang, China

**Keywords:** cross-sectional study, disease progression, gastric disease, *Helicobacter pylori*, longitudinal study

## Abstract

**Background:**

*Helicobacter pylori* (*H. pylori*) is a primary etiological agent for various gastric pathologies. Although numerous studies have described the association between *H. pylori* and individual stages of the Correa cascade, data comprehensively evaluating its prevalence across the full histopathological spectrum-from non-atrophic gastritis (NAG) to gastric cancer (GC)-within a single Chinese cohort remain limited. This study aimed to investigate the association between *H. pylori* infection and specific endoscopic stages, and further analyzed its influence on histopathological progression in a longitudinal sub-cohort.

**Methods:**

A retrospective study was conducted using data from participants who underwent concurrent gastroscopy and a ^14^C-Urea breath test (UBT) at the endoscopy center at Fengqing people’s Hospital between January 2019 and December 2024. Gastric mucosa status was assessed based on endoscopic and histopathological biopsy findings. Statistical associations were evaluated using the chi-square test. Correlations were analyzed using Spearman coefficients. A longitudinal sub-cohort was analyzed to compare disease progression between baseline *H. pylori*-positive and -negative groups. The hazard ratio (HR) and 95% confidence interval (CI) for incident disease progression were calculated by Cox proportional hazards regression.

**Results:**

A total of 13,579 participants were included, and the overall prevalence of *H. pylori* infection was 62.54%. A statistically significant association was found between *H. pylori* infection and gastric sequential stages of Correa’s cascade. The infection rate varied across pathological groups, being highest in participants with low-grade intraepithelial neoplasia (LGIN) (77.46%), and lowest in those with non-atrophic gastritis (NAG) (61.05%). In the longitudinal sub-cohort, the disease progression rate was higher among participants who were *H. pylori*-positive at baseline compared to their negative counterparts (9.67% vs. 5.60%, p = 0.0043). The HR for disease progression was significantly increased among H. pylori-positive participants compared with H. pylori-negative participants after sex- and age-adjustment [adjusted HR = 1.60 (1.07-2.39), p=0.021].

**Conclusion:**

Our study reveals a significant association between *H. pylori* infection and advanced gastric lesions, and confirms its significant impact on disease progression. This evidence solidifies the importance of eradication in clinical management to halt disease advancement.

## Introduction

Helicobacter pylori (*H. pylori*) is a gram-negative, microaerophilic bacterium that colonizes the human gastric mucosa. Since its discovery by Marshall and Warren in 1984, it has been recognized as a principal etiological agent in the pathogenesis of numerous gastric diseases (Marshall and Warren, 1984; Pan et al., 2024; Gisbert, 2025). Chronic infection with *H. pylori* is a primary driver of a spectrum of pathologies, ranging from chronic gastritis and peptic ulcer disease to gastric mucosa-associated lymphoid tissue lymphoma and, most critically, gastric cancer (GC) (Marshall and Warren, 1984; Xiao and Ma, 2022; Mulder et al., 2024). Consequently, the International Agency for Research on Cancer (IARC) has classified *H. pylori* as a Group 1 carcinogen (Moller et al., 1995).

The prevalence of *H. pylori* varies by geography, socioeconomic status, and age. Globally, *H. pylori* infects approximately half of the world’s population, with a disproportionate burden in low- and middle-income regions where infection remains a major public health challenge (Li et al., 2023). In China, the national prevalence is estimated at 42.8%, yet this figure masks profound regional disparities (Xie et al., 2024). Epidemiological data reveal a stark divide between urban (47%) and rural (66%) areas, highlighting that specific, high-risk populations are not adequately represented by national averages (Nagy et al., 2016). Fengqing County, a mountainous and underdeveloped rural region in Yunnan Province, exemplifies such a high-risk setting. Local cancer registry data identify GC as the leading malignancy in this area, yet a critical knowledge gap exists regarding the contemporary epidemiology of *H. pylori* in this adult population (Huang et al., 2024). Despite this clear disease burden, comprehensive, contemporary epidemiological data on *H. pylori* infection in the adult population of Fengqing County are scarce. This knowledge gap impedes the formulation and implementation of targeted public health interventions for *H. pylori* eradication and GC prevention.

The pathogenic sequence from *H. pylori*-induced inflammation to GC is canonically described as the “Correa cascade”, a multi-step model that unfolds over decades (Correa, 1992; Yeoh and Tan, 2022). This sequence involves a histological progression from chronic non-atrophic gastritis (NAG) to chronic atrophic gastritis (CAG), intestinal metaplasia (IM), gastric intraepithelial neoplasia (GIN), and ultimately invasive carcinoma. While *H. pylori* is the established initiator of this cascade, data on its prevalence at each distinct step of Correa’s cascade, and its variable influence on the risk of progression, are limited.

Elucidating the natural history of disease and quantifying progression risks requires longitudinal observation, as cross-sectional studies provide only static snapshots of disease prevalence. While pivotal longitudinal cohorts from Japan and Korea have established the risk of progression in *H. pylori*-infected individuals (Uemura et al., 2001; Kim et al., 2008), data from other high-risk populations, particularly from diverse regions within China, remain scarce.

Therefore, this study was designed to establish the contemporary prevalence of H. pylori in Fengqing County and to characterize its cross-sectional associations with endoscopic findings. Furthermore, leveraging a unique longitudinal sub-cohort, we sought to directly compare the progression rates of gastric diseases, between individuals with and without baseline H. pylori infection. This comprehensive investigation in a high-risk, underserved population aims to inform targeted public health strategies and deepen our understanding of H. pylori-driven carcinogenesis.

## Materials and methods

### Ethics statement

The study was approved by the Ethics Committee of Fengqing People’s Hospital, and had been performed in line with the ethical standards in the Declaration of Helsinki. Written informed consents were obtained from all participants in this study.

### Study population

A retrospective study with both cross-sectional and longitudinal components was conducted at the endoscopy center, Fengqing People’s Hospital. For the cross-sectional analysis, we reviewed the electronic medical records of all participants aged 18 years and older who underwent both an upper gastrointestinal endoscopy and a (Uemura et al., 2001) ^14^C-UBT between January 2019 and December 2024. Data were extracted from the hospital’s electronic database. The following variables were collected for each participant: demographic information (age, sex), data of examination (Uemura et al., 2001), ^14^C-UBT result, and the final endoscopic diagnosis. For the longitudinal cohort, follow-up endoscopic reports were also retrieved.

### Inclusion and exclusion criteria

The eligibility requirements were as follows: 1) Aged 18 years and older; 2) No history of cancer, mental disorders, or any contraindication to endoscopy; and 3) Had completed of upper gastrointestinal endoscopy with valid examination results. Participants with a history of partial or total gastrectomy, those who had received H. pylori eradication therapy within the past half year, or those who had used proton pump inhibitors (PPIs), bismuth compounds, or antibiotics within the four weeks prior to the UBT were excluded to ensure test accuracy. In addition, we excluded participants who were diagnosed with other malignancies during follow-up period.

### Urea breath analysis

H. pylori infection status was assessed using the (Uemura et al., 2001) C−urea breath test ( (Uemura et al., 2001) ^13^C−UBT), which was the standard non−invasive test routinely available at our hospital during the study period. And its diagnostic performance is comparable to that of (Kim et al., 2008) C−UBT for clinical diagnosis in adults (Wang et al., 2021). The ¹^4^C-UBT (Zhonghe Headway Bio-Sci & Tech Co. Ltd., China) was utilized to assess the status of H. pylori infection. The participants swallowed a test capsule that contains urea tagged with radioactive carbon-14 with water on an empty stomach or 2 h after eating. The 14C labeled urea was detected using an H. pylori detector (Shenzhen Zhonghe Headway BIO-SCI & TECH, China). A reading with more than 50 disintegrations per minute (DPM) was classified as H. pylori positive.

### Upper endoscopy examination

Subjects were required to abstain from food intake for 6 h before undergoing the endoscopic procedure. Gastroscopy was performed by a trained and qualified gastroenterologist with over five years of experience observed, documented, and evaluated the endoscopic images and biopsies. Because NAG is very common in people and typically not considered at increased risk for GC, participants diagnosed with NAG and normal gastric mucosa were grouped as the NAG group in this study. In addition, high-grade GIN (HGIN) and GC were combined into GC group (Pimentel-Nunes et al., 2019; Nagtegaal et al., 2020). Endoscopic findings were categorized by experienced gastroenterologists into one of the following groups: NAG, CAG, IM, low-grade GIN (LGIN), and GC.

### Longitudinal sub-cohort and definition of progression

From the main cohort, participants were eligible for the longitudinal sub-cohort if they underwent at least one follow-up gastroscopy at our hospital a minimum of six months after their baseline examination. Participants diagnosed with malignancies other than GC were excluded. This selection process yielded a longitudinal sub-cohort of 1,733 participants. The mean follow-up duration for this group was 28.26 ± 15.58 months.

Disease progression was defined as an advancement to a more severe histological stage on follow-up endoscopy. The progression was assessed along the recognized stages of the Correa cascade: from NAG to CAG, IM, LGIN, or GC. For instance, a participant with a baseline diagnosis of NAG who was subsequently diagnosed with CAG or IM was categorized as having experienced disease progression.

### Statistical analysis

The count data was presented using frequencies and rates (%), and statistical comparisons between groups were conducted using the chi-square test, Fisher’s exact probability method, and the continuity correction test. The measurement data was presented as mean ± standard deviation, and group comparisons were conducted using t-tests. Spearman correlations were computed to examine the underlying relationships between different groups and infection rates. HR with corresponding 95% CI for disease progression between the two groups were analyzed using the Cox proportional hazard regression model. The cumulative incidence curves between *H. pylori*-positive group and *H. pylori*-negative group using the Kaplan–Meier method and tested the differences using the log-rank test. All statistical analyses were performed using RStudio software (version 4.3.1). Statistically significant in all statistical analyses was defined as p <0.05 in a two-tailed test.

## Results

### Demographic and clinical characteristics

**Figure 1:**
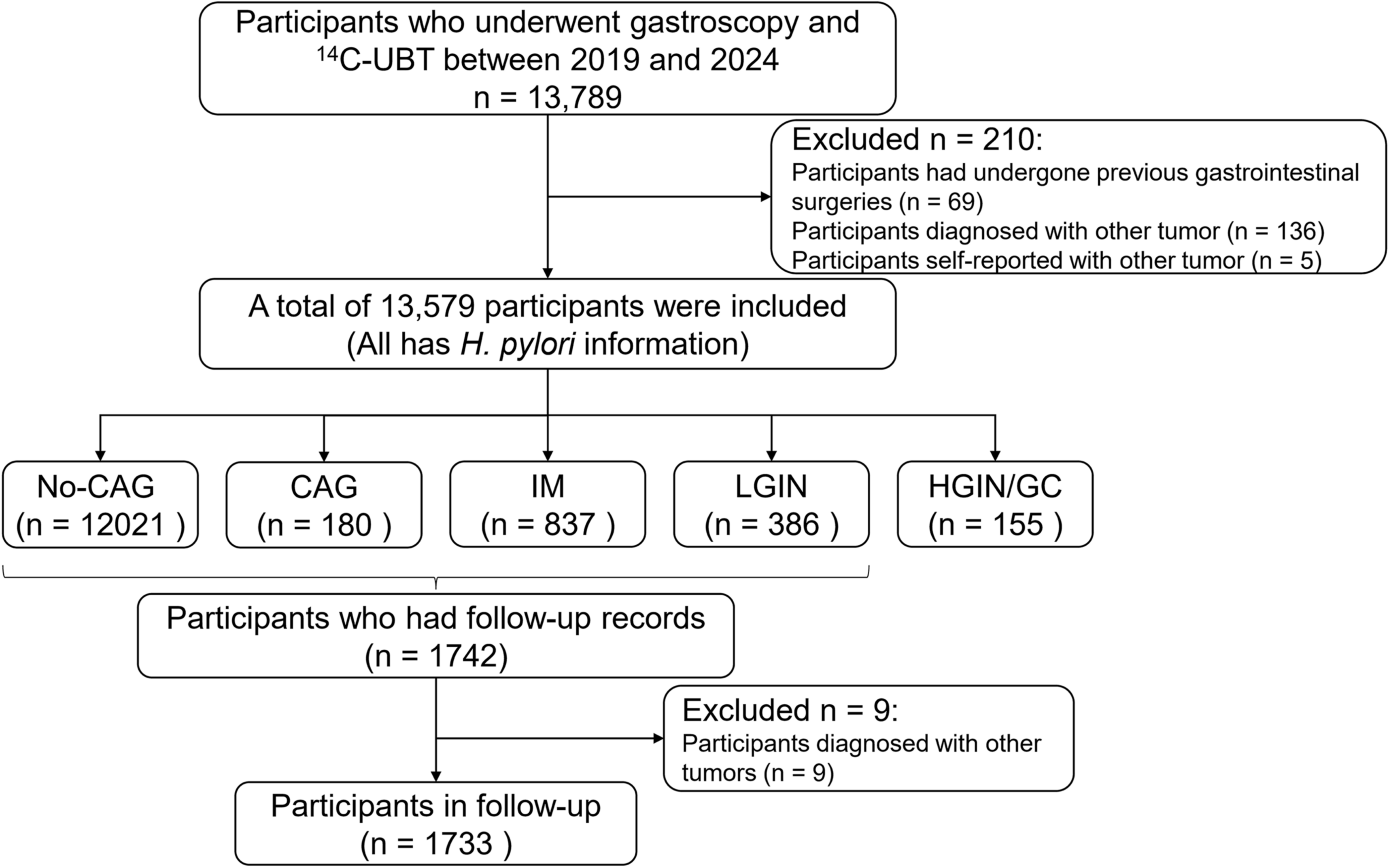
Flowchart of the study.

The process of enrolling participants in the study is depicted in **Figure 1**.

Based on the inclusion and exclusion criteria, a total of 13,579 participants were eligible for the cross-sectional analysis, and the overall prevalence of *H. pylori* infection in the cross-sectional cohort was 62.54% (8,492/13,579). Of these, a sub-cohort of 1,733 individuals with available follow-up data were included in the longitudinal analysis. The detailed baseline demographic and clinical characteristics of the cohort are presented in **Table 1**.

**Table 1:** Baseline characteristics and endoscopic findings of the study population.

Characteristic	Total (N = 13,579)	Longitudinal (N = 1,733)	Lost to follow-up (N = 11846)	p-value*
Age, mean (SD)	48.95 (11.98)	49.63 (11.41)	48.86 (12.07)	0.0129
Male, n (%)	6,143 (45.24)	806 (46.51)	5337 (45.05)	0.2554
H. pylori positive, n (%)	8,492 (62.54)	1,179 (68.03)	7313 (61.73)	<0.0001
Baseline pathology stage, n (%)				<0.0001
Non-Atrophic Gastritis (NAG)	12,021 (88.58)	1,512 (87.25)	10,509 (88.71)	
Chronic Atrophic Gastritis (CAG)	180 (1.33)	14 (0.81)	166 (1.40)	
Intestinal Metaplasia (IM)	837 (6.16)	129 (7.44)	708 (6.00)	
Low Gastric Intraepithelial Neoplasia (LGIN)	387 (2.85)	78 (4.50)	309 (2.61)	
High-grade Intraepithelial Neoplasia (HGIN)/Gastric Cancer (GC)	154 (1.13)			

*p-value comparing longitudinal vs. lost-to-follow-up (chi-square or t-test).

In the cross-sectional cohort, the mean participant age was 48.95 ± 11.98 years (median, 50; range, 25-84). The cohort comprised 6,143 males (45.24%) and 7,436 females (54.76%). The most common endoscopic diagnosis was NAG, observed in 88.58% of participants (n=12,021).

### Prevalence and distribution of *H. pylori* infection

**Figure 2:**
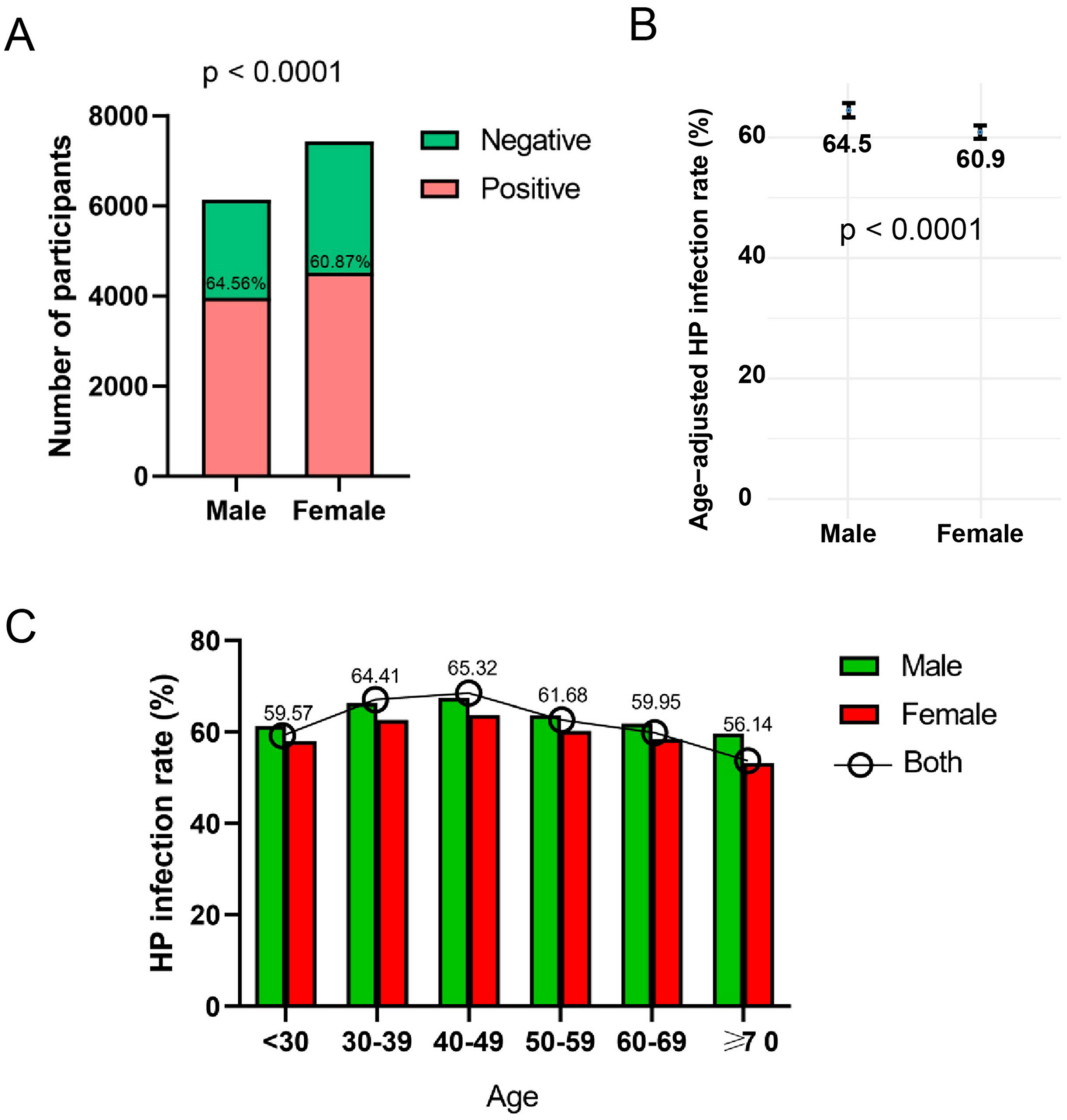
*H. pylori* infection status in participants of different sex and different age groups. **(A)** *H. pylori* infection status in participants of different sex. **(B)** *H. pylori* infection status in participants of different sex after age-adjustment. **(C)** *H. pylori* infection status in participants of different age groups.

First, we compared the sex differences of *H. pylori* infection rate. As shown in **Figure 2A**, the infection rates were significantly higher in males than in females (64.56% vs 60.87%; χ^2^ = 19.61; p < 0.0001). After adjusting for age using multivariable logistic regression, male sex remained significantly associated with a higher odds of *H. pylori* infection (OR = 1.17, 95% CI: 1.09-1.25, p < 0.0001). The age-standardized prevalence (marginal prediction) was 60.9% (95% CI: 59.8–62.0%) in females and 64.5% (95% CI: 63.3–65.7%) in males (**Figure 2B**). We further analyzed the age distribution and found the infection rate was statistically significant among different age groups (both: χ2 = 34.23, p< 0.0001; male: χ2 = 14.50, p= 0.0127; female: χ2 = 20.12, p= 0.0012) (**Supplementary Table 1**). Before the age of 49, the infection rate in males and females showed an increasing trend with the increase in age, and after the age of 49, it showed a decreasing trend (**Figure 2C**). Prevalence peaked at 65.32% in the group aged 40–49 years before declining to its lowest point of 56.14% in individuals aged 70 years and older. The detailed characteristics sex and age distributions of *H. pylori* infection are presented in **Supplementary Table 1**.

### Annual trend of H. pylori prevalence

**Figure 3:**
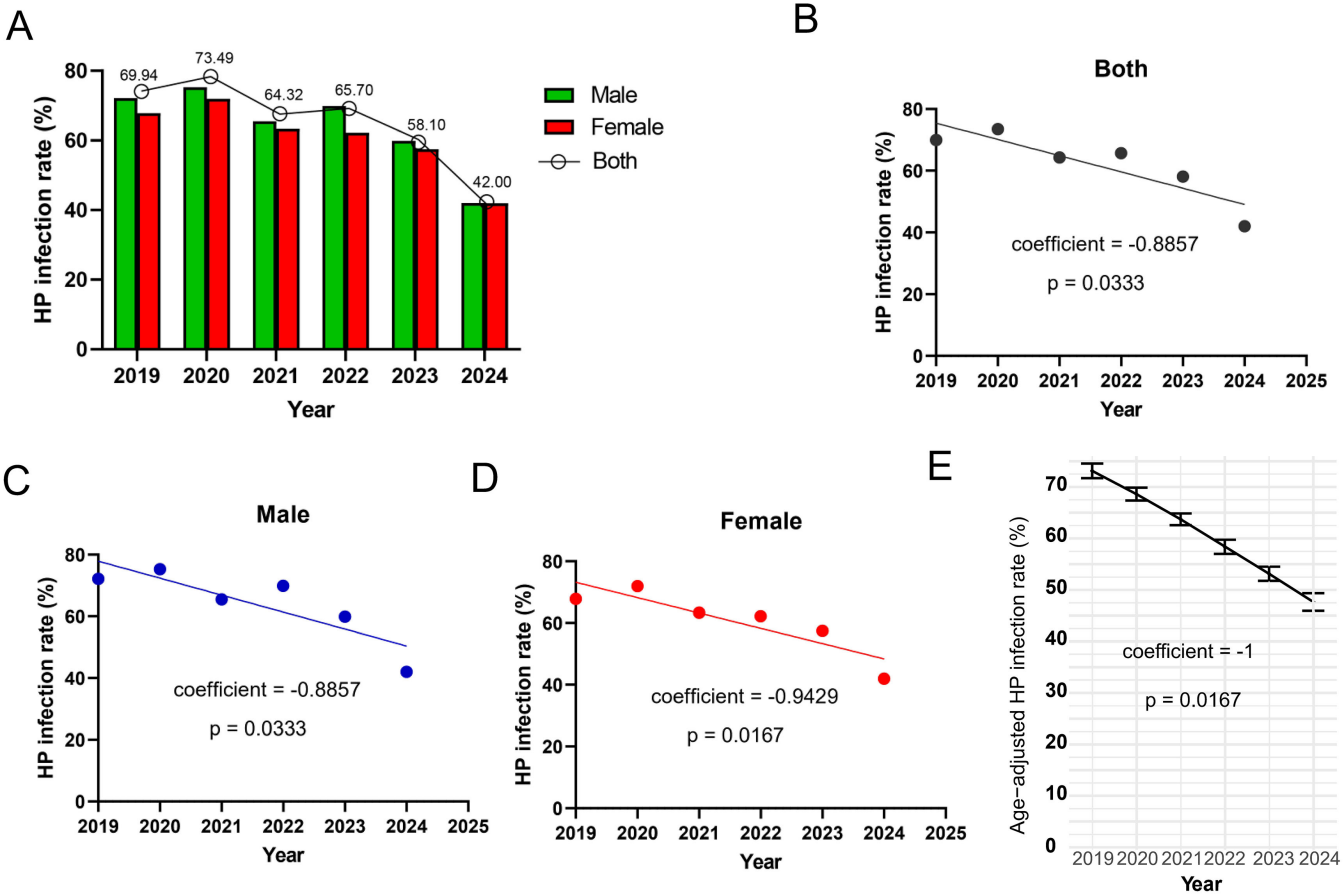
The trend of *H. pylori* prevalence over time. **(A)** *H. pylori* infection status in participants over time. **(B)** Spearman correlation between year and *H. pylori* prevalence in all participants (ρ = -0.8857, p = 0.0333). **(C)** Spearman correlation between year and *H. pylori* prevalence in male participants (ρ = -0.8857, p = 0.0333). **(D)** Spearman correlation between year and *H. pylori* prevalence in female participants (ρ = -0.9429, p = 0.0167). **(E)** Spearman correlation between year and *H. pylori* prevalence in all participants after age-adjustment (ρ = -1, p = 0.0167).

Next, we analyzed the annual prevalence of *H. pylori* infection. Over the six-year study period, a consistent and significant decline in *H. pylori* prevalence was observed (χ² = 588.6, p < 0.0001) (**Supplementary Table 2**). As shown in **Figure 3A**, the annual infection rate decreased from 69.94% in 2019 to 42.00% in 2024. We further calculated Spearman’s correlation coefficient (ρ) to evaluate the trend of *H. pylori* prevalence over time. For all participants combined, prevalence was significantly negatively correlated with year (ρ = -0.8857, p = 0.0333) (**Figure 3B**). Sex−stratified analysis revealed similar negative correlations in males (ρ = -0.8857, p = 0.0333) and females (ρ = -0.9429, p = 0.0167) (**Figures 3C, D**). After age−adjustment using logistic regression with marginal prediction, the negative trend remained statistically significant (Spearman’s ρ = -1, p = 0.0167) (**Figure 3E**). The detailed characteristics year distributions of *H. pylori* infection are presented in **Supplementary Table 2**.

### Association between *H. pylori* infection and upper gastrointestinal diseases

A strong association was identified between *H. pylori* infection status and the specific points in Correa’s cascade (χ² = 101.7, p < 0.0001). As shown in **Table 2**, *H. pylori* prevalence was markedly elevated in those with precancerous lesions compared to individuals with NAG (61.05%). Specifically, the highest rates of infection were observed in participants with LGIN (77.26%). Compared with NAG, the *H. pylori* prevalence in all other pathological states were significantly higher, with all p values < 0.05. We also calculated Spearman’s correlation coefficient between *H. pylori* prevalence and the sequential stages of Correa’s cascade. Although an increasing trend was observed, no significant correlation was found (**Supplementary Figure 1**). After age- and sex-adjustment using multivariable logistic regression, *H. pylori* prevalence was markedly elevated in those with precancerous lesions compared to individuals with NAG (**Table 2**).

**Table 2:** Association between *H. pylori* infection and endoscopic diagnosis.

Baseline pathology stage	Crude OR (95% CI)	Adjusted OR* (95% CI)	p-value
NAG	1 (ref.)	1 (ref.)	
CAG	1.71 (1.23-2.39)	1.72 (1.25-2.42)	0.0012
IM	1.77(1.51-2.07)	1.81 (1.55-2.13)	<0.0001
LGIN	2.17 (1.71-2.77)	2.20 (1.73-2.82)	<0.0001
GC	1.50 (1.07-2.14)	1.59 (1.13-2.28)	0.009

*Adjusted for age.

NAG, non-atrophic gastritis; CAG, chronic atrophic gastritis; IM, intestinal metaplasia; LGIN, low-grade intraepithelial neoplasia; GC, gastric cancer. OR, odds ratio; CI, confidence interval.

### Longitudinal analysis of disease progression

The longitudinal sub-cohort comprised 1,733 participants (1,179 *H. pylori*-positive, 554 *H. pylori*-negative) who were followed for a mean duration of 28.26 ± 15.58 months. Overall, disease progression was observed in 145 (8.37%) participants. The rate of progression was significantly different between the two groups. The rate of progression was significantly higher in the *H. pylori*-positive group (9.67%; 114/1,179) compared to the *H. pylori*-negative group (5.60%; 31/554; p = 0.0043) (**Table 3**).

**Table 3:** Association between *H. pylori* infection and disease progression.

Variables	Total (n= 1733)	Baseline H. pylori Status		
		H. pylori-positive (n=1179)	H. pylori-negative (n=554)	p-value
Age (years)	49.63 ± 11.41	49.13 ± 11.16	50.70 ± 11.88	0.0042
Sex				0.13
Male (n, %)	806 (46.51%)	563 (47.75%)	243 (43.86%)	
Female (n, %)	927 (53.49%)	616 (52.25%)	311 (56.14%)	
Duration of Follow-up (months)	28.26 ± 15.58	28.65 ± 15.52	27.43 ± 15.70	0.0816
Endoscopic Diagnosis				0.0219
NAG (n, %)	1512 (87.25%)	1010 (85.66%)	502 (90.61%)	
CAG (n, %)	14 (0.81%)	12 (1.02%)	2 (0.36%)	
IM (n, %)	129 (7.44%)	95 (8.06%)	34 (6.14%)	
LGIN (n, %)	78 (4.50%)	62 (5.26%)	16 (2.89%)	
Patients with Progression (n, %)	145 (8.37%)	114 (9.67%)	31 (5.60%)	0.0043
Patients with Stable/No Progression (n, %)	1588 (91.63%)	1065 (90.33%)	523 (94.40%)	

NAG, non-atrophic gastritis; CAG, chronic atrophic gastritis; IM, intestinal metaplasia; LGIN, low-grade intraepithelial neoplasia; GC, gastric cancer.

**Figure 4:**
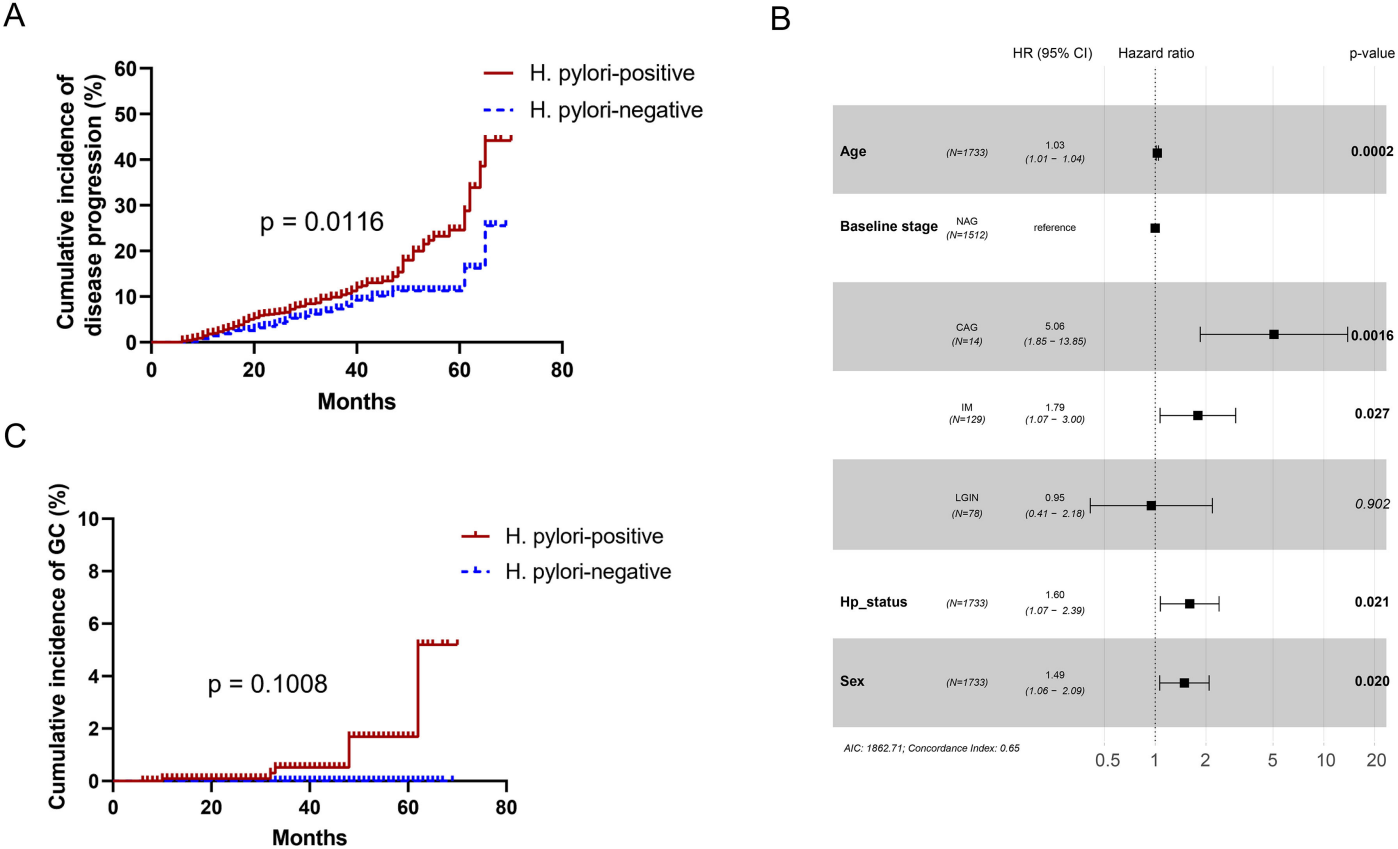
Comparison of cumulative incidence curves of disease progression in *H. pylori*–positive and *H. pylori*–negative group. **(A)** The cumulative incidence curves of disease progression in all participants (p=0.0116). **(B)** HRs and 95% CIs are presented for each variable included in the multivariable model. The model adjusted for age, sex, and baseline stage. *H. pylori* positivity (HR = 1.60, 95% CI: 1.12–2.39, p = 0.021), higher baseline stages (CAG, IM), and male sex were independently associated with faster progression. Error bars represent 95% confidence intervals. **(C)** The cumulative incidence curves of GC developed in all participants (p=0.1008).

Kaplan-Meier analysis confirmed that baseline *H. pylori* infection was a significant predictor of a higher cumulative incidence of disease progression (HR = 1.655, 95% CI = 1.164-2.353, p=0.0116) (**Figure 4A**). After adjustment, baseline *H. pylori* positivity was associated with a faster progression (adjusted HR = 1.60, 95% CI: 1.07–2.40, p = 0.021). Age, sex, and baseline stage also showed independent effects (**Figure 4B**). Critically, all incident cases of GC occurred exclusively within the *H. pylori*-positive cohort. During the follow-up period, GC developed in 7 of the 1,179 infected participants (0.59%) (**Table 4**). In contrast, no cases of GC were observed among the 554 uninfected participants. However, Kaplan-Meier analysis difference did not achieve statistical significance (p=0.1008) (**Figure 4C**).

**Table 4:** Details of the 7 cases in which gastric cancer developed.

Baseline stage	Hp baseline	Age/Sex	Follow-up Period (months)	Cancer type	Cancer site
LGIN	Positive	60/Male	62	AGC	Antrum
LGIN	Positive	55/Male	10	EGC	Angle
NAG	Positive	67/Female	48	EGC	Angle
LGIN	Positive	63/Male	32	EGC	Antrum
LGIN	Positive	61/Male	48	EGC	pylorus
LGIN	Positive	54/Male	19	EGC	Body
LGIN	Positive	54/Male	33	EGC	Antrum

NAG, non-atrophic gastritis; LGIN, low-grade intraepithelial neoplasia; AGC, Advanced gastric cancer; EGC, Early gastric cancer.

## Discussion

This large-scale, population-based study provides a comprehensive epidemiological and longitudinal assessment of *H. pylori* infection and its associated gastric pathologies in a rural Chinese population. Our cross-sectional analysis reaffirms the strong association between *H. pylori* infection and severe gastric pathologies, with prevalence rates of 77.26% in LGIN and 61.05% in NAG. This is consistent with established literature recognizing *H. pylori* as a key etiological agent (Uemura et al., 2001; Wang et al., 2014; Oh et al., 2025). Our longitudinal finding confirms that *H. pylori* infection is a powerful engine for disease progression, with infected individuals showing a significantly higher rate of disease progression over time. Thus, it provides a valuable guide for enhancing the diagnostic and therapeutic capabilities of primary care physicians in managing H. pylori infection.

*H. pylori* infection remains a major globally public health concern, with approximately 43.1% of adults infected, particularly in regions such as China (Li et al., 2023; Xie et al., 2024). Declining trends are observed in many developed countries, attributed to improved sanitation and widespread use of eradication therapies. However, the infection rate remains higher in rural areas than in urban areas (Cheng et al., 2009; Nagy et al., 2016; Ito et al., 2022). Given that understanding the epidemiology in rural areas holds greater significance, we conducted a detailed epidemiological analysis of *H. pylori* infection in Fengqing County, a rural region. Our observed overall *H. pylori* prevalence of 62.64% is consistent with rates reported in other developing regions, particularly within China, reaffirming its status as a major public health challenge (Li et al., 2023; Xie et al., 2024). Encouragingly, we documented a modest but steady annual decline in prevalence from 2019 to 2024. This trend likely reflects broader public health successes, including improvements in sanitation and hygiene, increased health awareness, and the implementation of effective eradication therapies, mirroring patterns seen in more developed nations (Li et al., 2023).

The demographic analysis revealed two key patterns. First, the observed higher prevalence in males is consistent with the global male predominance reported in systematic reviews and meta-analyses (de Martel and Parsonnet, 2006; Moshkowitz et al., 2012; Ibrahim et al., 2017). However, the magnitude of this sex difference varies across populations, and some studies have not identified a significant male predominance, reflecting the influence of regional, social, and cultural factors. Notably, a large Korean cohort study reported that adult men had a higher seroconversion rate from H. pylori−negative to positive than women during follow−up (Jung et al., 2013), indicating that men are more susceptible to incident infection. This Korean finding parallels our observation and suggests that true biological susceptibility may exist beyond age or behavioral confounders. If corroborated in additional prospective cohorts, this sex difference could inform targeted screening strategies in high−burden populations. The biological mechanisms underlying this potential increased susceptibility in men remain incompletely defined. Differences in lifestyle factors, occupational exposures, or health-seeking behaviors have been implicated (Ibrahim et al., 2017). In this study, our observation that the male predominance was attenuated after adjusting for age and other covariates does not refute a genuine biological difference, but rather highlights the importance of comprehensive confounding control. Nevertheless, confirmation in larger, well−powered prospective studies is needed. Second, we observed a characteristic inverted U-shaped prevalence curve with respect to age, peaking in the 40–49 year age group before declining in older individuals, which is consistent with previous study (Tsang et al., 2022). This complex pattern likely results from the interplay of two phenomena. One explanation involves a combination of a birth-cohort effect-whereby older generations had higher acquisition rates in youth due to poorer sanitation-and a survival bias, where individuals with severe, long-term *H. pylori*-related complications have higher mortality and are thus underrepresented in the oldest age groups. A more plausible explanation for this decline may be attributed to the cumulative incidental antibiotic exposure over time (even without formal *H pylori* eradication therapy) likely contributes by reducing the bacterial burden.

The relationship between *H. pylori* infection and gastric diseases has been extensively studied. *H. pylori* infection could cause histological, chronic and active inflammation, and the inflammatory response will be reduced after *H. pylori* eradication (Megraud et al., 2015; Yang et al., 2021; Malfertheiner et al., 2023; Wang et al., 2024). Consistent with this, our analysis revealed that *H. pylori* positivity rates increased in parallel with the severity of gastric lesions along the pre-neoplastic cascade (**Table 2**). Our results show that high infection rates were observed in all other pathological states compared with NAG. However, compared with patients with precancerous lesions, those with GC did not show an increased positivity rate; instead, a slight decrease was observed, although this difference was not statistically significant. This decline may be attributed to the natural history of the infection: chronic *H. pylori* infection induces extensive CAG and IM, which progressively render the gastric environment inhospitable and ultimately lead to spontaneous bacterial clearance (Sung et al., 2020). Indeed, spontaneous clearance rates of 40-80% have been reported in patients with advanced gastric pathology (Byun et al., 2025). This phenomenon can lead to an underestimation of the true lifetime prevalence of *H. pylori* in the elderly and may explain why some participants with “*H. pylori*-negative” GC may have had a past, cleared infection that initiated the carcinogenic process.

Although the rate of disease progression was higher among *H. pylori*–positive individuals, a non−negligible proportion of *H. pylori*–negative participants also experienced progression. Several factors may account for this observation. First, prior *H. pylori* infection followed by spontaneous clearance or undocumented eradication could lead to a negative UBT despite prior mucosal damage that predisposes to subsequent progression. Second, false−negative test results-resulting from, for instance, recent antibiotic use or low bacterial load-may lead to misclassification. Third, gastric carcinogenesis can proceed via *H. pylori*–independent mechanisms, such as high-nitrate diet, genetic predisposition, or dysbiosis of non−*H. pylori* gastric microbiota (Fu et al., 2024; Ding et al., 2026; He et al., 2026; Jiang et al., 2026). Finally, baseline histopathological changes present prior to the index test could also influence later progression.

The strength of our study is significantly enhanced by the longitudinal analysis, which moves beyond static association to illuminate causality. The finding that *H. pylori*-positive individuals had a significantly higher rate of disease progression (9.67% vs. 5.60%; p =0.0043) provides powerful prospective evidence that the bacterium is an active driver of pathological advancement. Consistent with the findings of Kim et al. from Korea, our results further validate the Correa cascade model. In their 9.4−year follow−up of a large Korean cohort, all five incident GC cases were *H. pylori*−positive, and GC developed 10.9 times more frequently in the presence of baseline IM than in its absence. Consistent with these observations, all seven GC cases identified in our longitudinal cohort also arose from *H. pylori*−positive participants, with six of the seven having a baseline diagnosis of LGIN. This striking concordance across two distinct East Asian populations underscores the critical role of H. pylori infection as an initiating event in gastric carcinogenesis, while simultaneously highlighting LGIN as a particularly high−risk precursor stage that warrants close surveillance. Most critically, all incident cases of GC during follow-up occurred exclusively in the *H. pylori*-positive group. This starkly illustrates the natural history of the infection in the absence of intervention and powerfully reinforces the clinical rationale for “test-and-treat” strategies to halt progression towards malignancy (Megraud et al., 2015; Malfertheiner et al., 2023; Pan et al., 2024; Ford et al., 2025).

This study has several strengths. The large sample size of the cross-sectional analysis provides robust prevalence data. Critically, the inclusion of a longitudinal sub-cohort adds a prospective dimension, strengthening our conclusions regarding disease progression. Third, all participants were enrolled from a homogenous, census-registered population, which minimizes confounding from genetic diversity. Nonetheless, certain limitations must be acknowledged. First, although our study included a large number of participants, as a single-center study, our findings may have limited generalizability, and multicenter validation is warranted. Second, differential follow−up should be considered when interpreting the longitudinal cohort findings. Participants who completed at least one additional endoscopy had a higher baseline *H. pylori* prevalence and a greater proportion of advanced gastric lesions (such as IM and LGIN) compared with those lost to follow−up. This likely reflects clinical practice patterns, where individuals with more severe symptoms or known precancerous conditions are more motivated to adhere to endoscopic surveillance, whereas those with milder disease or negative *H. pylori* status may be less inclined to return. Consequently, the observed progression rates may be influenced by this imbalance. Nevertheless, the prospective nature of follow−up and the comprehensive adjustment for baseline covariates help to mitigate potential bias. Future studies with standardized surveillance protocols are warranted to further validate our findings. Third, we did not perform strain characterization of *H. pylori* (e.g., CagA or VacA genotype), as infection status was determined non−invasively by (Uemura et al., 2001) ^14^C-UBT, and gastric biopsies or bacterial isolates were not available for molecular typing. Given the well-established role of strain virulence factors in modulating disease phenotype and progression risk (Ohnishi et al., 2008; Whitmire et al., 2025), the absence of genotype data constitutes an important limitation of this study. Future prospective investigations should incorporate endoscopic biopsy with culture or PCR−based methods, or validated serologic markers, to evaluate the contribution of specific virulence factors to disease progression. Finally, our study lacked data on *H. pylori* eradication treatment and therapeutic outcomes during the follow-up period, precluding a direct assessment of treatment effects. However, the efficacy of eradication in reducing GC incidence is already well-established by extensive evidence from randomized controlled trials (Pan et al., 2024; Ford et al., 2025). Further larger cohorts with multicenter studies are, therefore, required to address these limitations.

In conclusion, this study demonstrates that *H. pylori* infection is significantly associated with advanced gastric lesions, and is a confirmed driver of disease progression, solidifying the imperative for eradication in clinical management. Collectively, these findings underscore the critical importance of *H. pylori* eradication as a primary strategy to halt disease progression and prevent gastric cancers.

## Data Availability

The raw data supporting the conclusions of this article will be made available by the authors, without undue reservation.

## Supplementary material

Supplementary Figure 1 Pearman's correlation coefficient between *H. pylori* prevalence and the sequential stages of Correa's cascade.
