# Supplementary figures and images for: Association of Helicobacter pylori infection with the correa cascade: a single-center, retrospective cohort study
Source: Front Cell Infect Microbiol. 2026 Jul 8;16:1838434. doi: 10.3389/fcimb.2026.1838434 (PMC13388916; doi:10.3389/fcimb.2026.1838434)

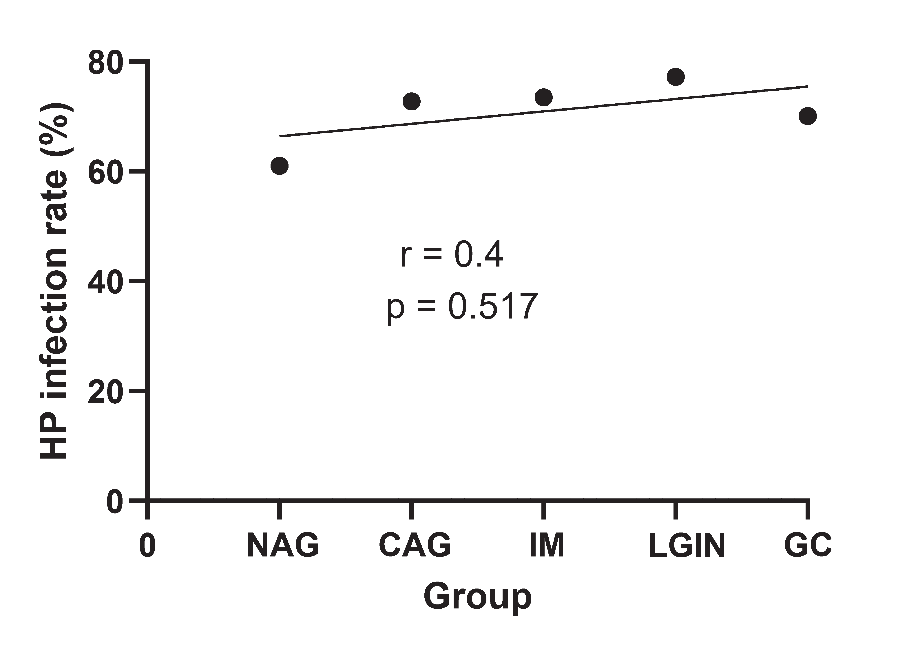

Supplement: Supplementary Figure 1 — Pearman's correlation coefficient between H. pylori prevalence and the sequential stages of Correa's cascade. [file Image1.png]
