# Supplementary material for: Association of Helicobacter pylori infection with the correa cascade: a single-center, retrospective cohort study
Source: Front Cell Infect Microbiol. 2026 Jul 8;16:1838434. doi: 10.3389/fcimb.2026.1838434 (PMC13388916; doi:10.3389/fcimb.2026.1838434)
Supplement: Supplementary file 2 [file DataSheet1.docx]

Supplementary Table 1. Sex and Age Distributions of *H. pylori* Infection.

| **Age Group (years)** | **Male and female** | | | **Male** | | | **Female** | | |  |  |  |  |
| --- | --- | --- | --- | --- | --- | --- | --- | --- | --- | --- | --- | --- | --- |
|  | n | **%** | **Infection rate, %** | n | **%** | **Infection rate, %** | n | **%** | **Infection rate, %** | **OR** | **95% CI** | **χ2** | **p-value** |
| <30 | **705** | 5.19 | 59.57 | **341** | 5.55 | 61.29 | **364** | 4.9 | 57.97 | 1.148 | 0.845 - 1.194 | 0.8074 | 0.3689 |
| 30-39 | **2,672** | 19.68 | 64.41 | **1,285** | 20.92 | 66.30 | **1,387** | 18.65 | 62.65 | 1.173 | 1.002- 1.374 | 3.877 | 0.0489 |
| 40-49 | **3,397** | 25.02 | 65.32 | **1,525** | 24.83 | 67.34 | **1,872** | 25.17 | 63.68 | 1.176 | 1.020 - 1.378 | 4.994 | 0.0254 |
| 50-59 | **4,135** | 30.45 | 61.69 | **1,775** | 28.89 | 63.66 | **2,360** | 31.74 | 60.21 | 1.158 | 0.510 -1.314 | 5.103 | 0.0239 |
| 60-69 | **2,116** | 15.58 | 59.97 | **962** | 15.66 | 61.85 | **1,154** | 15.52 | 58.4 | 1.155 | 0.969 - 1.377 | 2.593 | 0.1073 |
| ≥70 | **554** | 4.08 | 56.14 | **255** | 4.15 | 60.00 | **299** | 4.02 | 53.18 | 1.299 | 0.9249 - 1.834 | 2.311 | 0.1284 |
| **Total** | **13,579** | **100** | **62.54** | **6,143** | **100** | **64.56** | **7,436** | **100** | **60.87** |  |  |  |  |
| **χ2** |  |  | **34.23** |  |  | **14.50** |  |  | **20.12** |  |  |  |  |
| **p-value** |  |  | **0.0001** |  |  | **0.0127** |  |  | **0.0012** |  |  |  |  |

Values with (%) are frequencies.

CI, confidence interval; OR, odds ratio.

Supplementary Table 2. Temporal Trend of *H. pylori* Prevalence.

| **Year** | **Crude prevalence (%)** | **Age‑adjusted prevalence (%)** | **95% CI** |
| --- | --- | --- | --- |
| 2019 | 69.94 | 74.37 | 73.14 – 75.60 |
| 2020 | 73.49 | 69.97 | 69.95 – 70.99 |
| 2021 | 64.32 | 65.17 | 64.31 – 66.01 |
| 2022 | 65.77 | 62.2 | 61.40 – 63.00 |
| 2023 | 58.12 | 54.66 | 53.53 – 55.80 |
| 2024 | 42.00 | 49.19 | 47.66 – 50.71 |

Values with (%) are frequencies.

CI, confidence Interval
